# Research on the Mechanism of Soybean Resistance to *Phytophthora* Infection Using Machine Learning Methods

**DOI:** 10.3389/fgene.2021.634635

**Published:** 2021-02-11

**Authors:** Junxia Chi, Shizeng Song, Hao Zhang, Yuanning Liu, Hengyi Zhao, Liyan Dong

**Affiliations:** ^1^College of Software, Jilin University, Changchun, China; ^2^Key Laboratory of Symbolic Computation and Knowledge Engineering of Ministry of Education, Jilin University, Changchun, China; ^3^College of Computer Science and Technology, Jilin University, Changchun, China

**Keywords:** sRNA data analysis, differential expression, machine learning, resistance mechanism, *Phytophthora sojae*

## Abstract

Since the emergence of the *Phytophthora sojae* infection, economic losses of 10–20 billion U.S. dollars have been annually reported. Studies have revealed that *P. sojae* works by releasing effect factors such as small RNA in the process of infecting soybeans, but research on the interaction mechanism between plants and fungi at the small RNA level remains vague and unclear. For this reason, studying the resistance mechanism of the hosts after *P. sojae* invades soybeans has critical theoretical and practical significance for increasing soybean yield. The present article is premised on the high-throughput data published by the National Center of Biotechnology Information (NCBI). We selected 732 sRNA sequences through big data analysis whose expression level increased sharply after soybean was infected by *P. sojae* and 36 sRNA sequences with massive expression levels newly generated after infection. This article analyzes the resistance mechanism of soybean to *P. sojae* from two aspects of plant’s own passive stress and active resistance. These 768 sRNA sequences are targeted to soybean mRNA and *P. sojae* mRNA, and 2,979 and 1,683 targets are obtained, respectively. The PageRank algorithm was used to screen the core functional clusters, and 50 core nodes targeted to soybeans were obtained, which were analyzed for functional enrichment, and 12 KEGG_Pathway and 18 Go(BP) were obtained. The node targeted to *P. sojae* was subjected to functional enrichment analysis to obtain 11 KEGG_Pathway. The results show that there are multiple Go(BP) and KEGG_Pathway related to soybean growth and defense and reverse resistance of *P. sojae*. In addition, by comparing the small RNA prediction model of soybean resistance with *Phytophthora* pathogenicity constructed by the three machine learning methods of random forest, support vector machine, and XGBoost, about the accuracy, precision, recall rate, and F-measure, the results show that the three models have satisfied classification effect. Among the three models, XGBoost had an accuracy rate of 86.98% in the verification set.

## Introduction

Soybean is an indispensable high-protein food, healthy vegetable oil, and raw material for essential health products. Widely planted across the globe and having a history spanning 5,000 years of cultivation, soybeans are integral in agricultural products. As a vital basic material related to the national economy and people’s livelihood, soybean is the most economically beneficial crop. Notably, the demand for soybean in China is about 110 million tons per year, while the output of soybeans is about 17 million tons. Thus, increasing soybean production is a significant issue linked to the livelihood of people.

Root rot is one of the primary diseases of soybeans (Ranathunge et al., 2008). Often appearing and causing harm during the entire growth cycle of soybeans, this disease will cause seed rot before emergence, plant wilt after emergence, and leaf wilting and yellowing during adult plant stage and will cause rot and stunted growth and so on (Dorrance, 2018). Every year, root rot caused by *Phytophthora sojae* is estimated to incur economic losses of more than 1 billion U.S. dollars in soybean production worldwide (Kaufmann and Gerdemann, 1957). Determining how to reliably prevent root rot has always been an extensively discussed research issue. In particular, as the soybean planting area has continued to increase in recent years, research on this issue has quickly become urgent. Hence, in the present study, a new perspective was adopted in exploring the resistance mechanism of soybeans after being infected by *P. sojae*, and the effects of soybeans on themselves and *P. sojae* after infection were analyzed. The main reason for this was because study of durable and broad-spectrum control strategies suitable for soybeans is of considerable significance for increasing soybean production and income.

Since the first experimental discovery of sRNA in 1993, sRNA research has made a considerable amount of progress, and researchers have progressively devoted themselves to sRNA research (Brodersen and Voinnet, 2006; Pertea et al., 2016; Li and Li, 2018). sRNA interferes with the standard translation of RNA through complementary base pairing with mRNA and even degrades mRNA to cause the silencing thereof, thereby affecting the protein expression level of organisms and having a regulatory function in the growth and development of organisms (Gottesman, 2005; Huang et al., 2009; Attila et al., 2010; Aucher et al., 2013; Chen et al., 2020). In addition to having the previously mentioned regulatory function in the organism itself, recent research has demonstrated that sRNA can also enter other species through filming and other methods to maintain activity and control other species’ life process (Deng et al., 2018). For example, Zhang et al. (2011) discovered that miR168a in plant rice can enter animals through ingestion and inhibit the standard translation process of mRNA that translates LDLRAP1 protein in animal liver tissues, as well as achieve cross-species regulation of animals. Research by Zhou et al. (2015) verified that plant miRNAs can stably exist in the lungs of experimental mice and inhibit the self-replication of influenza A virus.

In recent years, studies have determined that sRNA can be utilized as an effector to be transmitted between interacting pathogenic bacteria and plants and can regulate the expression level of target mRNA according to the RNA interference mechanism. This mechanism is referred to as a cross-species regulatory mechanism (Weiberg et al., 2015). For instance, Arne W et al. discovered in 2013 that when *Botrytis cinerea* infects tomatoes, sRNA molecules are released, thereby invading the tomatoes, inducing silencing of genes related to tomato immunity, and achieving an inhibitory effect on the host plant’s immune process (Altuvia, 2007; Weiberg et al., 2013). Weiberg et al. (2015) confirmed through experiments that the sRNA of *B. cinerea* can silence the mRNA of *Arabidopsis* translating AGO1 protein by pairing and binding, thereby affecting the normal immune response of *Arabidopsis*. *Arabidopsis* sRNA can also be transmitted to the pathogen *B. cinerea* and inhibit the expression of pathogenicity-related genes of the pathogen *B. cinerea*, as found by Cai et al. (2018). The aforementioned research offers a new direction and further ideas for plant disease research.

There are no on the construction of sRNA prediction models concerning soybean resistance to *P. sojae* infection combined with machine learning. Research on the possibility of reverse regulation of *P. sojae* by soybeans from the sRNA level remains in the infancy stage, while the analysis of sRNA remains at the stage of biological experiments. There is no report on the data analysis method of combining computer methods to study plant resistance to fungal infection. In the present article, the differentially expressed sRNA sequences of soybeans infected by *P. sojae* were adopted as the data basis. Construction of an sRNA prediction model for soybean resistance to *Phytophthora* pathogenicity was deemed as being of major significance for predicting the disease resistance potential of unknown soybean sRNA and its close-source sRNA. At the same time, the sRNA sequences were respectively targeted to soybean and *Phytophthora* and predicated on the PageRank algorithm to mine the core regulatory modules, analyze the function of the regulatory pathway, and then verify the role of the selected soybean disease resistance key sRNA sequence in resisting *Phytophthora* disease. The present article is innovative in studying the possible regulatory effects of soybean on autoimmune response and *P. sojae* in sRNA. Meanwhile, the present paper provides a data basis for the research on the interaction mechanism of soybean and *P. sojae*, a theoretical basis for increasing soybean yield and income, and a data analysis plan for the research on other plant fungi interactions, having vital scientific significance.

## Data and Methods

**Figure 1:**
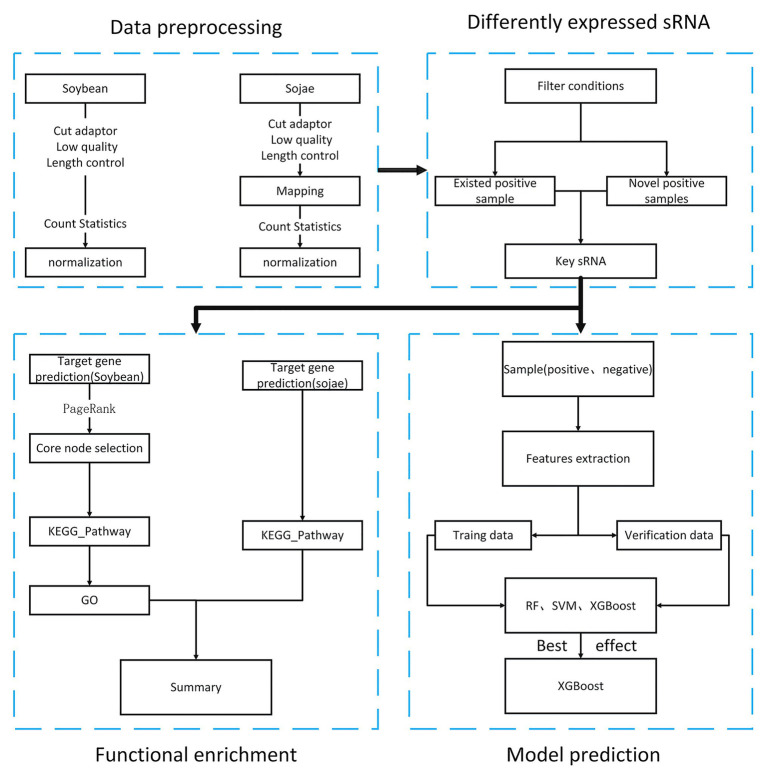
Research flowchart of soybean resistance mechanism.

Pursuant to the National Center of Biotechnology Information (NCBI) public database, the aim of the present article was to obtain soybean sRNA data before and after being infected by *Phytophthora sojae*, and through preprocessing such as removal of joints, removal of low-quality data, and standardization. After statistical analysis, the differentially expressed soybean sRNA data before and after infection by *P. sojae* were acquired. First, by targeting the differentially expressed soybean sRNA to the mRNA data of soybean and *P. sojae*, the target mRNA was analyzed for functional enrichment, and the biological processes and functional pathways related to the defense of passive and active resistance in soybean to infection were discovered. In addition, by comparing the sRNA prediction model of soybean resistance with *Phytophthora* pathogenicity constructed by the three machine learning methods, a finding was that XGBoost had a better effect. The overall process of this article is revealed in Figure 1.

### Data Source and Preprocessing

#### Data Sources

In the present article, data were obtained from NCBI for samples with the same culture conditions except for infection, namely, soybean sRNA infected by *P. sojae* and blank samples (GSM1370294) for soybeans that were not infected by *P. sojae*. The data included *P. sojae* genome data (*P. sojae* V3.0), soybean genome data (Wm82.gnm1), *P. sojae* mRNA data (W05.gnm1), and soybean mRNA data (Wm82.gnm1.ann1).

#### Joint Treatment

**Figure 2:**
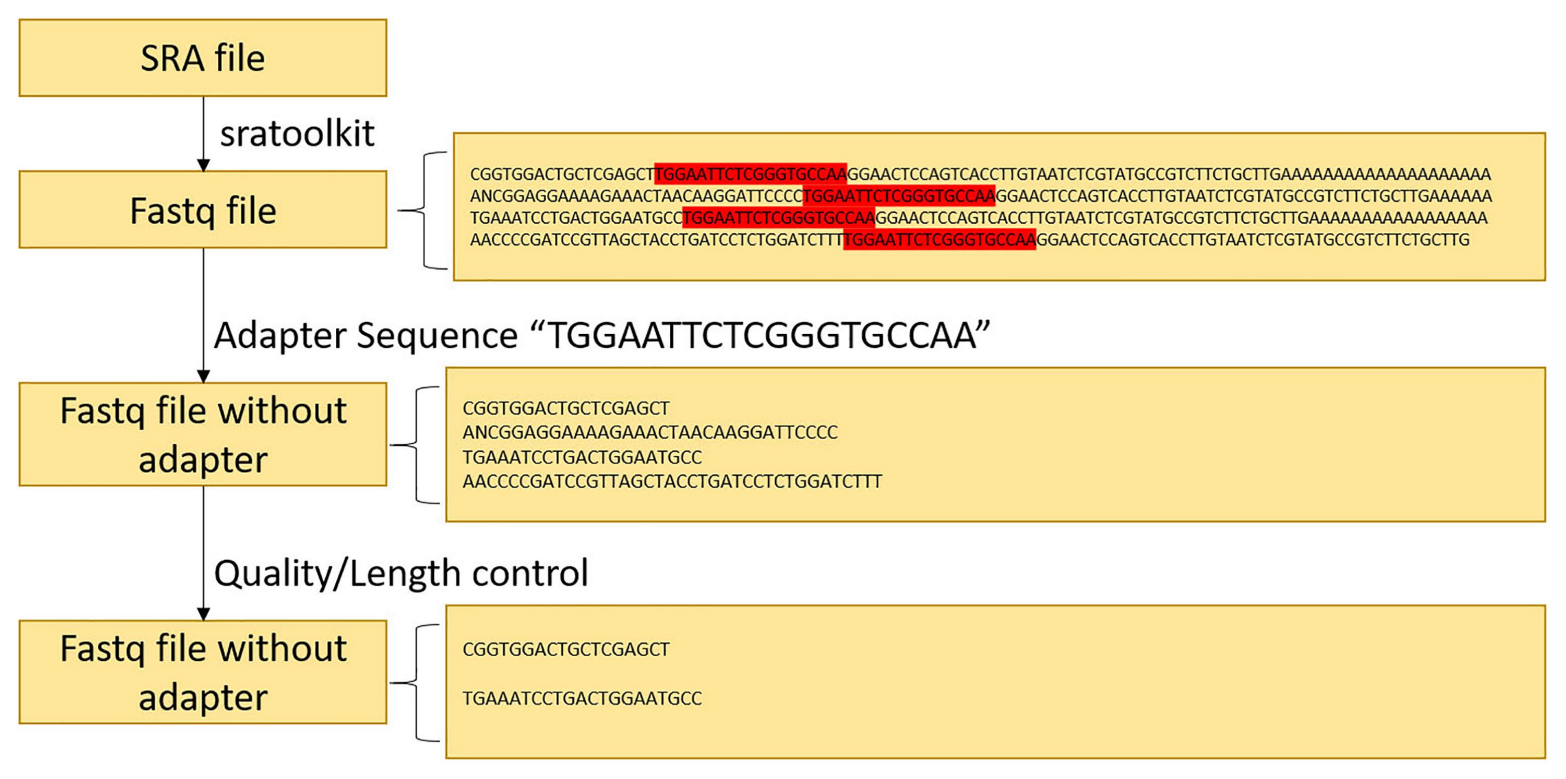
Data preprocessing flowchart.

Since the data downloaded on NCBI are in SRA format, the tools of sratoolkit.2.10.4^1^ were adopted in the present article for conversion to FASTQ format. After conversion, the sRNA sequence of the same length was obtained. In the vein of obtaining the correct actual sequence, removal of the linker was necessary. The linker sequence was found to be “TGGAATTCTCGGGTGCCAA” after consulting. To obtain valid data without redundant information, the Cutadapter^2^ was employed to remove the joints of the experimental group of soybeans infected by *P. sojae* and the blank group sequence not infected by *P. sojae*. The joint processing process is shown in Figure 2.

#### Quality Information and Length Processing

**Figure 3:**
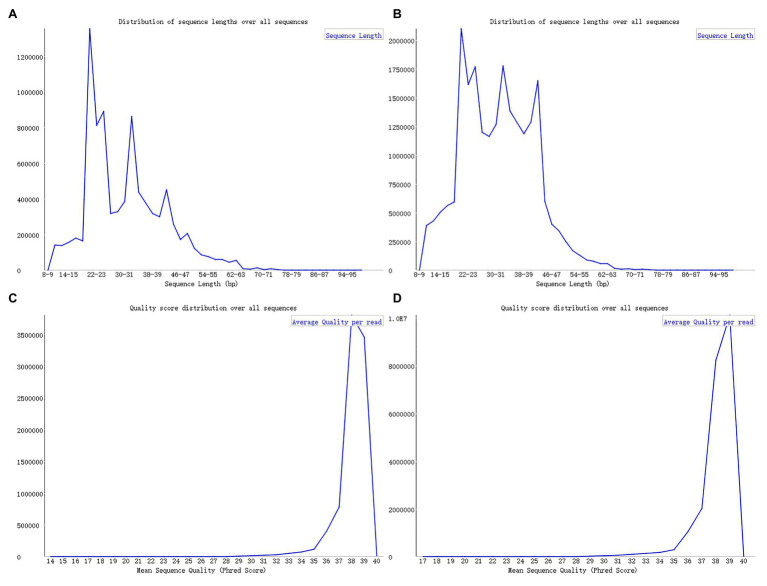
FastQC sequence evaluation results. **(A)** The sRNA data of the blank group of soybeans without quality control. **(B)** The sRNA data of the experimental group infected by *Phytophthora sojae* without quality control. The abscissa in the figure signifies the sequence length and the ordinate indicates the number of sequences. **(C,D)** The corresponding quality control results. The ordinate represents the number of sequences, while the abscissa denotes the average base quality of the sequences.

Upon analyzing the data after removing the joints, an observation could be made that the original data had not been operated on length, quality control, etc. The specific length and the sequence number distribution corresponding to each length are presented in Figures 3A,B. Considering the limitations of instruments, equipment, and difficulty in condition control during biological experiments, certain errors in the data can easily occur. To ensure the rigor of the experiment, FastQC^3^ was employed in the present article to control the sequence quality. Here, at least 80% of the bases in each sRNA sequence to were set to have a quality value greater than or equal to 33. The length distribution diagrams obtained after controlling the quality are exhibited in Figures 3C,D. The present article assumes that the length of the sRNA sequence that can target soybean and *P. sojae* genes should be 18–25 nt, only sRNA sequences of 18–25 nt are retained. Upon removing joints and removing low quality and length control, sequence expression statistics were respectively performed on the blank group soybean sRNA data; and the experimental group sRNA data infected by *P. sojae*, the specific method, were the data de-duplicated to obtain the sequence type, and the number of each sequence in the statistical data file was the expression level of the sequence. The quantity statistics of the two groups of sRNA are reported in Table 1, where soybean is the blank group of soybean sRNA sequence, and infected mixed data are the sRNA sequence of the experimental group after infection.

**Table 1:** Data volume statistics before and after quality control.

Raw data	After adapter (not de-duplicated)	Length and quality control (not de-duplicated)	De-duplicated
Soybean	8,828,481	3,226,820	902,702
Infected mixed data	22,405,771	6,073,928	1,251,487

#### Data Mapping to the *Phytophthora sojae* Genome

**Figure 4:**
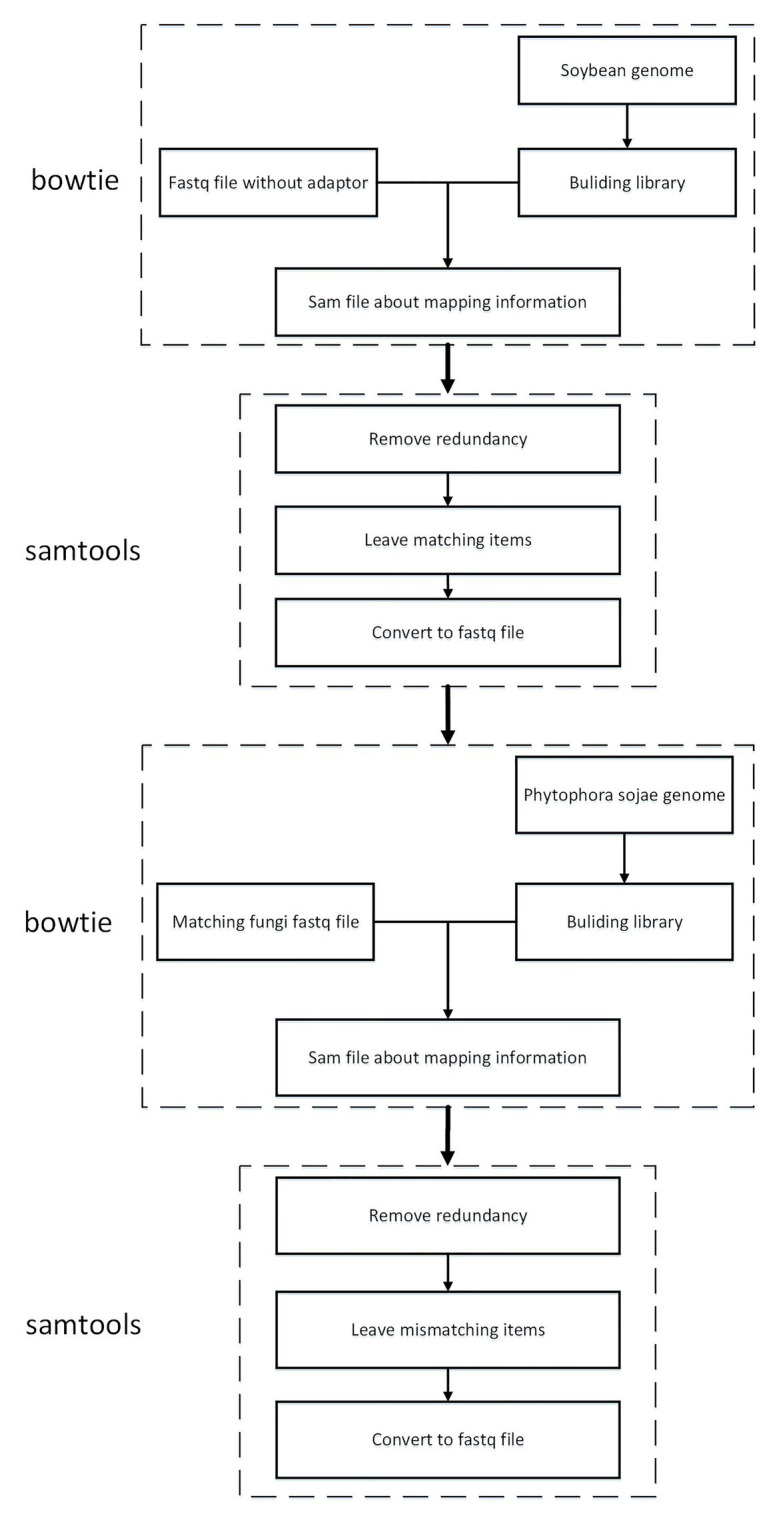
Roadmap for data mapping to genome technology.

To guarantee that the sRNA data belonged to soybean and not *P. sojae* or pollutants, the present article mapped the sRNA sequence infected by *P. sojae* to the soybean genome and retained the matched sequence. Subsequently, the obtained sequence was mapped to the *P. sojae* genome, with unmatched sequences being kept. Here, the present article used bowtie2-2.3.4.1,^4^ a tool for mapping short sequences to the genome, and samtools-1.9,^5^ a tool set for manipulating SAM and BAM, to map sequences to the genome, as can be observed in Figure 4. The specific steps included the following: (1) building a database based on the soybean genome to obtain an index file package for matching operations; (2) mapping the FASTQ file of the sRNA sequence infected by *P. sojae* and the fasta file of the blank sample sequence, using the bowtie tool to perform strict matching based on the index package of the first step, setting the mismatch parameter to 0 and keeping all the comparison results output to the SAM file; (3) using SAMtools to convert SAM files to BAM files; (4) performing de-redundancy and keeping matching sequences; (5) converting BAM files to FASTQ files; and (6) repeating the same as above to build a library of *P. sojae* genes, performing the above operation on the FASTQ data retained in the soybean genome, and retaining the data that do not match the *Phytophthora* soybean genome.

The present article was written based on the type of analysis of the small RNA sequence after *P. sojae* infection. Following statistics, after removal of the joints, quality information processing, and length control, but not yet mapping, the sequence types were 1,251,487. There were 1,112,350 kinds of small RNA sequences mapped to the soybean genome. There were 1,091,544 kinds of small RNA sequences that mapped to the soybean genome and could not be mapped to *P. sojae*. As revealed in Table 2, after this step, a determination could be made that the remaining 1,091,544 sRNAs belonged to soybean sRNA. Pursuant to the data of 1,091,544 kinds of sRNA, the expression changes before and after infection were compared and analyzed in the present paper, and the differentially expressed sRNA was screened.

**Table 2:** Data volume statistics before and after map.

Sequence category	Number of sequence types
Original infection sequence	1,251,487
Matches to soybean and does not match to *Phytophthora sojae* genome	1,091,544

### Data Standardization and Multiple Analysis of the Difference

An observation can be made from Table 1 that the overall expression and types of sRNA notably changed before and after infection. Thus, the commonly used normalization, Min-Max standardization, log function conversion, and anta function conversion were not suitable for standardizing current data. Here, the quartile standardization of the expression levels before and after infection was conducted to render the two comparable. By sorting all sequences in ascending order on the basis of the expression level and then selecting the three-quarter position as the reference point, the expression level was set to 1, while the expression levels of other sequences were converted into multiples of the expression level at the reference point. After this step, the expression levels of all sequences in the sample were converted into multiple relationships relative to the expression level of the sample’s reference point. This method can avoid the difference in base, type, and quantity between different samples.

The present paper compares the 1,091,544 sRNA sequences that match soybean and do not match the *P. sojae* genome with the blank group. A finding was that 192,283 species existed in the sequence before and after infection, and only 899,261 species existed in the sequence after infection. In terms of the 192,283 species that appeared before and after infection, the present paper assumes that after normalizing the data, the expression level after infection is significantly higher than the expression level before infection; that is, after the multiple increase, the sequence with higher expression level after infection is a positive sample that is integral in the soybean resistance mechanism. The specific calculation method is denoted in Eq. 1:


(1) $$\mathrm{Rate}=\frac{count(after)-count(before)}{count(before)}$$

**Figure 5:**
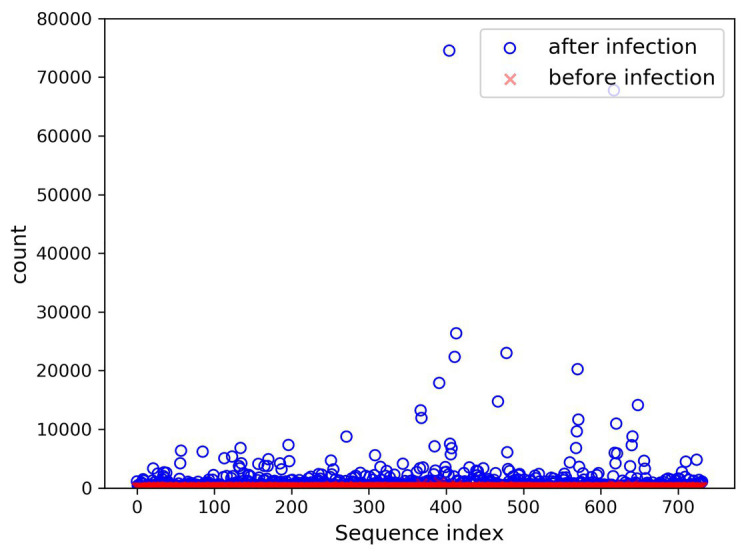
Seven hundred thirty-two changes in expression before and after infection.

**Figure 6:**
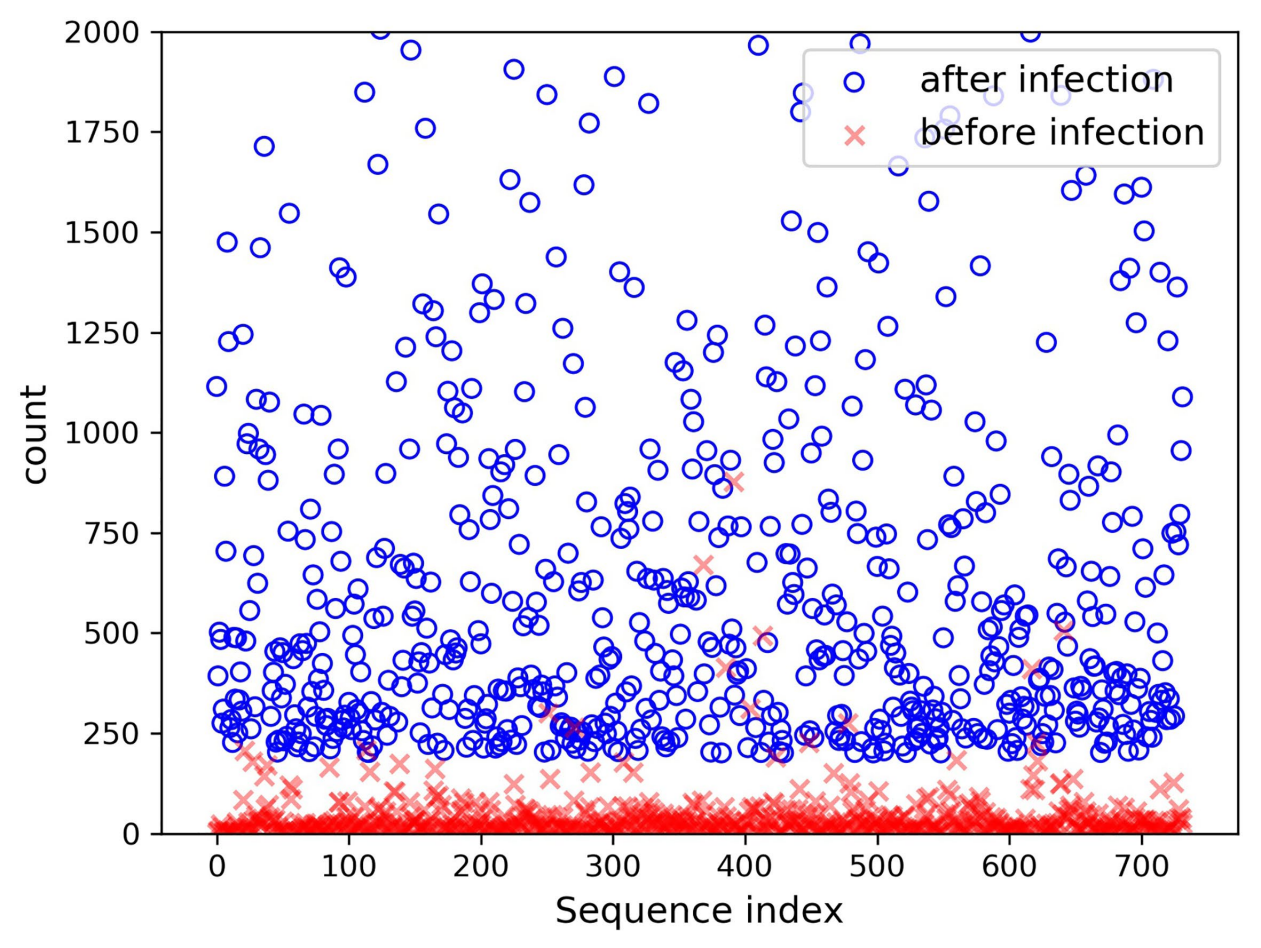
Changes in expression level (upper limit 2,000) before and after infection.

In the present paper, the positive samples were based on both the growth rate and the expression level, and the data after infection and mapping were screened. The first part is the sample shared by the infected group and the blank group that meets the growth rate higher than 10 and the expression level higher than 200. Two conditions resulted in 732 sequences. The difference in expression levels before and after infection is exhibited in Figure 5. The abscissa indicates the sequence index, which is sorted in lexicographic order, and the ordinate refers to the expression level. Owing to the excessive expression of individual positive samples, Figure 5 is not clear and intuitive. An observation can be made from the figure that most sequences’ expression quantity was less than 2,000. Hence, Figure 6 was drawn with the expression level of 2,000 as the upper limit, where the expression level of the same sequence was substantially different before and after infection. The second part is the newly generated sequence in the infection group, with the blank group having no corresponding sequence. Here, 36 sequences with expression levels higher than 100 were selected, and a total of 768 sequences were utilized as positive samples.

### Target Gene Prediction

Predicated on the 768 sequences selected above, in order to find the biological processes and regulatory pathways related to the soybean resistance mechanism, the target genes of soybean and *P. sojae* were respectively predicted. At this point, the Tapir-1.2^6^ was applied in the present article to make predictions. First, the file was converted to FASTA format, and all T bases were replaced with U bases, and then the target prediction was performed on the mRNA data of soybean and *P. sojae*, respectively, using the default parameters of the Tapir tool, namely, mimic = 0, score ≤ 4, mfe_ratio ≥ 0.7. The target gene prediction results were counted after running on the server, with the results indicating that a total of 2,979 types of soybean mRNA and a total of 1,683 types of *P. sojae* mRNA can be targeted.

### Select the Core Node That Targets the Soybean Itself

In the present paper, String database^7^ and SoyBase database^8^ were adopted for KEGG_Pathway regulatory pathway analysis and GO(BP) function enrichment analysis. Because of the large number of target prediction results for soybeans, the obvious enrichment pathways were not clear and intuitive. PageRank is a method used by Google to identify the rank/importance of a web page and is the only standard used by Google to measure the quality of a website. The algorithm is predominantly premised on two assumptions: first, if a node is pointed to by many nodes, the node is considered to be more important; and second, if the value of a node itself is relatively high, the node pointed to by the node is considered to be more important. The functional path enrichment network has similar features in function. If there are more nodes in the functional path pointing to a certain node, the node is often the main control function. In parallel, the general functionality of the node pointed to by the important node is also stronger. Therefore, in the present paper, the PageRank algorithm was utilized to screen the core function clusters. The specific calculation method is denoted in Eq. 2:


(2) $$\mathrm{PR}\left(a\right)=\overset{B_{a}}{\underset{b=B_{1}}{\sum }}\frac{PR\left(b\right)}{L\left(b\right)}$$

Among them, PR(a) refers to the PageRank value of the u-th node, B(a) signifies the set of incoming links of all a, and L(b) indicates the degree of all outgoing links of the v node pointing to the a node.

**Figure 7:**
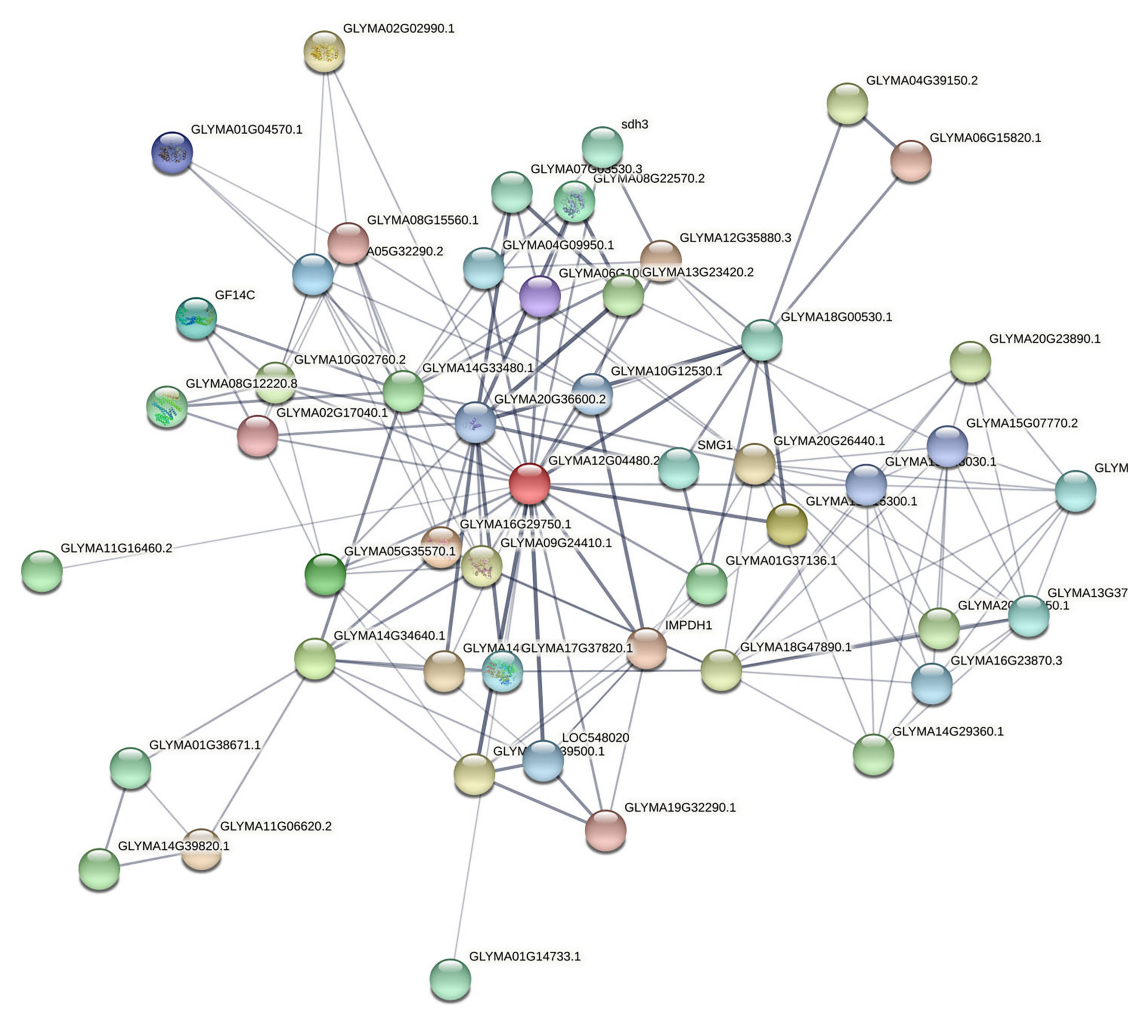
Core nodes targeting soybeans screened by PageRank algorithm. Here, the confidence line type was selected, where the nodes in the figure represent the proteins targeted to soybeans, and the connections between the nodes represent the interaction relationships between the proteins. Thickness indicates the strength of the interaction between proteins.

The network – based on the core function clusters – was analyzed again to select the more significant functions further. The core functional cluster regulatory network diagram of the selected 50 nodes is revealed in Figure 7.

### Predictive Model Construction

#### Negative Sample Selection

**Figure 8:**
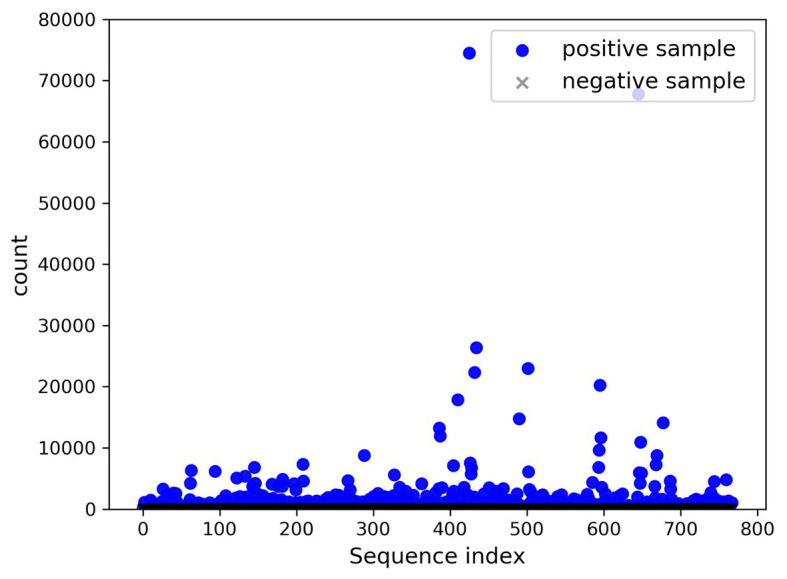
Comparison of positive and negative sample expression levels.

**Figure 9:**
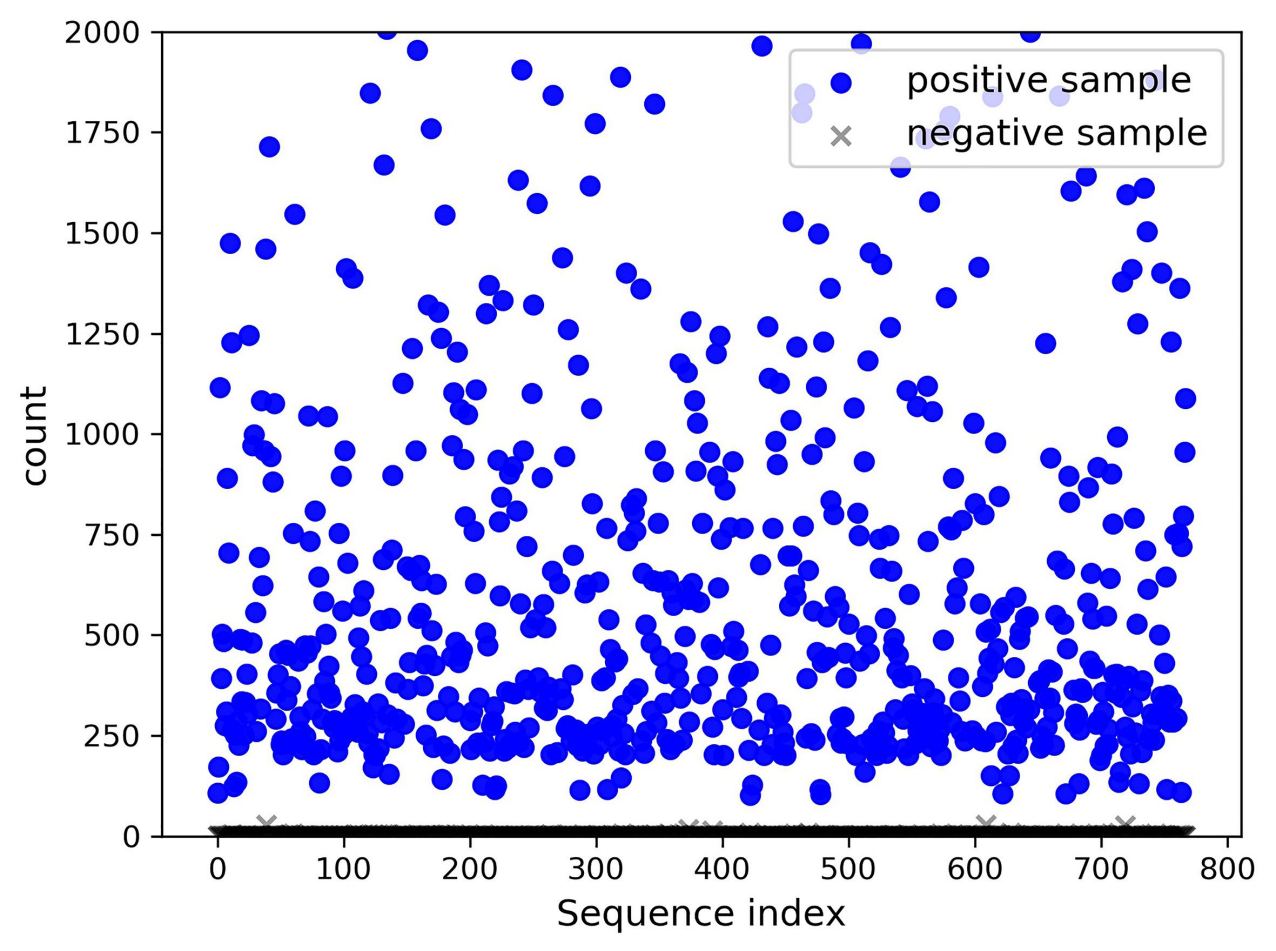
Comparison chart of positive and negative sample expression (upper limit 2,000).

**Figure 10:**
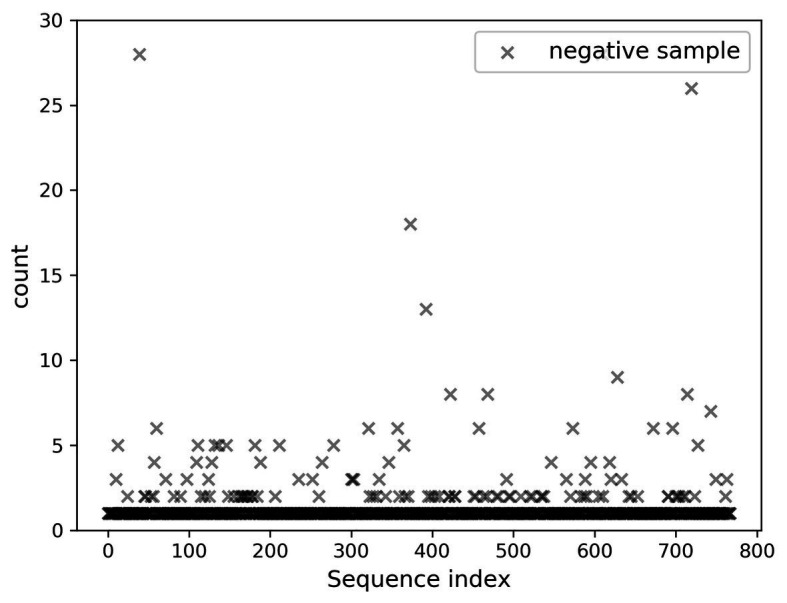
Display of negative sample expression.

A total of 768 differentially expressed sRNA sequences in 2.2 were selected as the positive samples in the present article. Then calculations were performed on the intersection of the blank group of soybean data and the experimental group data infected by *P. sojae* to obtain coexisting data. The sequences with a growth rate of 1 before and after infection were screened from the data, and then the same number of sRNA sequences as the positive samples was randomly selected as negative samples. The comparison chart of positive and negative sample expression is revealed in Figure 8, where the abscissa represents the sequence index, which is sorted in dictionary order, and the ordinate signifies the expression of the sequence. Since the expression of individual positive samples was too large, the image was blurred, but observation can be made that the nodes’ expression in the graph was mostly concentrated below 2,000. For this reason, Figure 9 was drawn as presented. A further observation can be made that the difference between the positive and negative samples was significant, but the negative sample was still not clear enough; thus, the negative sample expression map was drawn as shown in Figure 10.

#### Feature Extraction

Under the verified features and the analysis of the current sequence, the present paper proposed a total of 114 features including sequence features (sequence length and local base) and structural features [guanine-cytosine (GC)%, motif frequency (1–3 nt), and minimum free energy (MFE)]. Regarding the local base and 3'-end double base and 5'-end double base, the values were A, G, C, U, and N. If the length was less than 25 nt, N was used to substitute in addition to that. Since the features needed in machine learning were digital, one-hot encoding was performed for these sequence features. The specific encoding form is presented in Table 3. The RNAfold^9^ tool was adopted to acquire the MFE. The specific feature selection information, the number of each feature, the number of features after one-hot encoding, and the total number of features are listed in Table 4. These 203 features were used for subsequent operations.

**Table 3:** Codes corresponding to bases.

Base	One hot
N	0000
A	0001
G	0010
C	0100
U	1,000

**Table 4:** Features selected by prediction model.

Feature name	Feature number	One hot number
Local base	25	100
Sequence length	1	1
GC%	1	1
MFE	1	1
5'-end double base	1	8
3'-end double base	1	8
Motif frequency (1–3 nt)	84	84
Total	114	203

MFE, minimum free energy.

Local base: used to indicate the type of base at positions 1 to 25 in the sequence. The length of the sequence is less than 25 nt, and the insufficient part is filled with N. Sequence length: indicates the length of sRNA; here, it refers to 18–25 nt. GC%: The ratio of the number of occurrences of “G (guanine)” and “C (cytosine)” in the sequence to the total length of the sequence. GC content has a significant effect on the stability and structure of sRNA. MFE: The MFE of sRNA is an important measure of its structural stability. In this paper, the MFE is obtained using the RNAfold tool. Under normal circumstances, this indicator can measure the probability of interaction between RNAs and can be used as a standard to distinguish all coding and non-coding RNAs. 5'-end double base, 3'-end double base: Since DNA transcription and RNA translation have specific sequence and directionality, the first two bases at the 3'-end and 5'-end are used to mark the beginning of various processes. Motif (1–3 nt): small sequence fragments with specific functions that repeatedly appear in the sequence. The number of their appearances can play a certain role in determining the overall feature function of the sequence. Among them, 1 nt has four features, 2 nt has 16 features, and 3 nt has 64 features.

After the feature selection was completed, low-variance features were removed, and then subsequent model selection and training were performed. This was because there may have been redundant features in those mentioned above, which would affect the classifier’s prediction effect.

#### Selection of Model Algorithm

Based on sRNA’s characteristics, constructing a predictive model of soybean differentially expressed sRNA can reduce the cost of experimental research. Because this article is a prediction model constructed for high-dimensional, small-sample binary classification problems and does not have too many requirements for time and space complexity, three machine learning models, which are support vector machines (SVMs), random forests, and XGBoost, are used to deal with classification problems.

SVM is a binary classification technique based on supervised learning in machine learning. It is called the optimal edge classifier. It has good learning performance and has good performance in the classification of high-dimensional data. The effect is that it is not easy to overfit. It can effectively classify samples with small data volumes, avoid the problem of neural network structure selection and local minimum points, and have been widely used.

Random forest is a supervised learning algorithm, it is the most representative of ensemble algorithms, and it shows amazing performance in classification and regression. The basic idea of the algorithm is to construct multiple weak classifier decision trees by randomly extracting samples and features. Only when more than half of the base classifiers make mistakes will they make wrong predictions. It can handle high-dimensional data without feature selection (because the feature subset is randomly selected). Due to random sampling, the trained model has small variance and strong generalization ability and is not easy to overfit.

XGBoost is a scalable machine learning system for tree boosting. The system has been widely recognized in a large number of machine learning and data mining challenges due to its outstanding efficiency and high prediction accuracy. The XGBoost algorithm realizes the generation of a weak learner by optimizing the structured loss function (the loss function of the regular term is added, which can reduce the risk of overfitting), and the XGBoost algorithm does not use the search method but directly uses the loss function and the first-order and second-order derivatives of, and the presorting, weighted quantile and other techniques to improve the performance of the algorithm significantly.

#### Data Set Sample Division and Standardization

As mentioned in *Negative Sample Selection* section, there were 1,536 positive and negative samples. In the present article, the training set and the validation set were constructed according to the ratio of 3:1. Then, the training set data were employed for model training and parameter adjustment, and the validation set data were applied to evaluate the final trained model.

To ensure that all the features had a similar effect on the model, the data set needed to be standardized after the feature extraction, and then the standardized data set was utilized for model training. Because the present article’s model needed to use distance to measure similarity, and individual feature data have outliers, the Z_Score standardization method was adopted to process 203 feature data sets. The specific calculation method is as shown in Eq. 3:


(3) $${\mathrm{Feature}}_{\mathrm{new}}=\frac{\mathrm{Feature}‐\mu }{\sigma }$$

where μ is the mean of all sample data, *σ* is the standard deviation of all sample data, ${\mathrm{Feature}}_{\mathrm{new}}$ is the standardized data set, and Feature is the pre-standardized data set.

#### Model Cross-Validation to Select Optimal Parameters

The aforementioned divided data set was employed to construct a binary classification model using three methods: random forest, SVM, and XGBoost. The models were adjusted to the optimal parameters through cross-validation, so that each model had the best effect. For a two classifier, the evaluation indicators mainly included accuracy, recall, precision, and F1 value. The accuracy rate was used to calculate the correctly classified samples’ ratio in the classifier to the total samples. The specific calculation method is as exhibited in Eq. 4:


(4) $$\mathrm{Accuracy}=\frac{TP+TN}{TP+TN+FP+FN}$$

Evaluating an algorithm model solely on accuracy is far from being scientific and comprehensive. Under normal circumstances, the precision rate, recall and F1 value can better predict skew problems. Accuracy refers to the proportion of correct predictions that are positive to all positive predictions, while recall denotes the proportion of correct predictions that are positive to all actuals that are positive. The F1 value is evaluated by calculating the harmonic mean of precision and recall. The calculation methods for precision, recall, and the F1 value are as presented in Eqs. 5–7:


(5) $$\mathrm{Precision}=\frac{\mathrm{TP}}{\mathrm{TP}+\mathrm{FP}}$$

(6) $$\mathrm{Recall}=\frac{\mathrm{TP}}{\mathrm{TP}+\mathrm{FN}}$$

(7) $$F1=\frac{2PR}{P+R}=\frac{2TP}{2TP+FP+FN}$$

In the present article, TP refers to the number of positive samples classified correctly, TN signifies the number of negative samples classified correctly, FN indicates the number of positive samples classified as negative samples, and FP is the number of negative samples classified as positive samples.

Since the deviations of the data set in the present article have already been discussed in *Feature Extraction* section, accuracy was adopted as the main evaluation index, with recall, precision, and F1 being auxiliary evaluation indexes. The parameters of the three models were then accordingly selected. Because there are many parameters to be determined, the present paper utilized grid search combined with cross-validation to determine the optimal parameters. In the training process of SVM, the inner product kernel function could be used to replace the nonlinear mapping to the high-dimensional space. The final decision was only determined by a small number of support vectors. The overall complexity thereof depended on the number of support vectors and was not sensitive to abnormal data. Hence, the choice of the number of support vectors was integral in the overall effect of the model, and the gamma parameter was a parameter of the radial basis function (RBF) kernel function, and the specific value thereof was inversely proportional to the number of support vectors. Thus, in the present article, gamma and model error tolerance C are primarily determined.

Multiple base learners jointly determined the results of random forest and XGBoost, and the two parameters n_estimators and max_depth had a greater impact on the effects of these two models. n_estimators refers to the maximum number of base learners that need to be divided, and a compromise is needed to select the optimal number of base learners. This is because normally as the number of base learners increases, the model’s error rate will gradually converge, but the code complexity will gradually increase. max_depth denotes the maximum depth of the decision tree, where generally, the deeper the depth of the tree, the better the fit. Yet overfitting is common. The present article included a large number of features, and the selection of this value needed to be considered. In addition, the random forest also selects the max_features parameter, which indicates the maximum number of features considered when randomly selecting features and is equivalent to a decision tree when equal to the number of features. XGBoost also selects the gamma parameter and subsample parameter. The gamma parameter specifies the minimum loss function drop required for node splitting. The larger the value of this parameter, the more conservative the algorithm. The subsample parameter controls the random sampling ratio of each tree and does not put back sampling. Decrease the value of this parameter, and the algorithm will be more conservative and avoid overfitting. However, if this value is set too small, it may cause underfitting.

The specific process for the grid search for optimal parameters was as follows: (1) selecting 1,152 test set sequences as the sample set for parameter optimization; (2) determining the value range of the main parameters to be optimized. For SVM, C was 0.001–100, and gamma was 0.001–100; for the random forest, n_estimators was 1–102, max_depth was 3–14, and max_features was 3–11. For XGBoost, n_estimators was 10–100, max_depth was 2–14, subsample was (0, 1), and gamma was 0–100. (3) For each model, selecting different parameter values for combination, the specific process being using accuracy as a measurement index, using three-fold cross-validation for training and evaluation, and recording the parameter corresponding to the maximum score. (4) Repeating operations (2) and (3) above and selecting the optimal parameters corresponding to each model.

The optimal parameters determined by the grid search were as follows: for the SVM, after selecting the RBF kernel function, the optimal parameters were 0.25 for C and 0.01 for gamma; for the random forest algorithm, the optimal parameters were 91 for the number of base learners, 6 for the maximum depth, and 5 for the maximum number of features; for the XGBoost algorithm, the optimal parameters were 90 for the number of base learners, 2 for the maximum depth, 0.7 for subsample, and 0.001 for gamma.

## Results

### Functional Enrichment Analysis of Soybean sRNA Targeting Nodes

#### Functional Enrichment Analysis of Targeting Soybean Itself

In the present paper, these 50 core nodes in *Select the Core Node That Targets the Soybean Itself* section were again imported into the String database, and the results reveal that the overall protein-protein interaction (PPI) enrichment *p*-value of the regulatory network was 1.96e−10. Among them, the *p*-value of 12 KEGG_Pathway was less than the credibility threshold of 0.05, as shown in Table 5. The regulatory pathways exhibited in the table were sorted according to credibility. In addition to credibility, an observation can be made that the higher the number of genes in the pathway, the more significant the pathway’s effect.

**Table 5:** KEGG_Pathway of soybean to resist *Phytophthora* infection sRNA targeting soybean mRNA.

Pathway	Description	False discovery rate	Number of genes
gmx00230	Purine metabolism	0.00014	5
gmx04626	Plant-pathogen interaction	0.00014	5
gmx00240	Pyrimidine metabolism	0.00033	4
gmx01100	Metabolic pathways	0.00033	12
gmx03015	mRNA surveillance pathway	0.00033	4
gmx04141	Protein processing in endoplasmic reticulum	0.0012	4
gmx04136	Autophagy – other	0.0054	2
gmx03040	Spliceosome	0.0073	3
gmx03440	Homologous recombination	0.0082	2
gmx00562	Inositol phosphate metabolism	0.0126	2
gmx04070	Phosphatidylinositol signaling system	0.0126	2
gmx00480	Glutathione metabolism	0.0187	2

The role of soybean after being infected by *Phytophthora sojae* can be predominantly divided into three categories: regulating the metabolic level thereof to resist *Phytophthora* infection, regulating the sRNA production process, and changing the membrane permeability. Among the aforementioned regulatory pathways, the higher-ranking pathways included plant-pathogen interaction (false discovery rate was 0.00014), mRNA surveillance pathway (false discovery rate was 0.00033), and protein processing in endoplasmic (false discovery rate was 0.0012). This indicates that soybean sRNA can indeed directly resist the infection of *P. sojae*. Further, the phosphoinositide metabolism and phosphatidylinositol signaling system with a false discovery rate of 0.0126 can change cell membranes’ permeability, having crucial effects on the binding of *P. sojae* to the soybean cell membrane to release sRNA into the soybean cell.

To further study the specific biological function of soybean for self-regulation, the GO biological function was analyzed in the present paper premised on mature annotation files. Since the target for target gene prediction was inconsistent with the mRNA version in SoyBase, the annotation file(Glyma_11_to_Glyma_20_Correspondence_Full) provided by SoyBase was first used to convert the mRNA version, and GO(BP) enrichment analysis was subsequently performed. The results highlight that there were 18 *p*-values less than the credible threshold of 0.05, as presented in Table 6.

**Table 6:** Soybean resistance to *Phytophthora* infection sRNA targeting soybean GO enrichment results.

GO	Description	False discovery rate	Number of genes
GO:0008026	ATP-dependent helicase activity	5.55E−09	15
GO:0004386	Helicase activity	3.65E−08	16
GO:0006355	Regulation of transcription, DNA dependent	4.18E−07	4
GO:0003700	Sequence-specific DNA binding transcription factor activity	3.74E−06	3
GO:0008135	Translation factor activity, nucleic acid binding	6.41E−05	5
GO:0004430	1-Phosphatidylinositol 4-kinase activity	0.000563865	4
GO:0003746	Translation elongation factor activity	0.001187864	6
GO:0006259	DNA metabolic process	0.001424074	7
GO:0003824	Catalytic activity	0.00359907	3
GO:0007126	Meiosis	0.004201701	8
GO:0003676	Nucleic acid binding	0.015695101	26
GO:0000086	G2/M transition of mitotic cell cycle	0.019566168	2
GO:0042817	Pyridoxal metabolic process	0.019566168	2
GO:0045876	Positive regulation of sister chromatid cohesion	0.019566168	2
GO:0051304	Chromosome separation	0.019566168	2
GO:0048015	Phosphatidylinositol-mediated signaling	0.040720139	3
GO:0004004	ATP-dependent RNA helicase activity	0.04021511	3
GO:0016876	Ligase activity, forming aminoacyl-tRNA and related compounds	0.04668625	3

From Table 6, observations can be made on the regulation of transcription, DNA-dependent (false discovery rate was 4.18E−07), sequence-specific DNA binding transcription factor activity (false discovery rate was 3.74E−06), translation factor activity, nucleic acid binding (false discovery rate was 0.001187864), ATP-dependent RNA helicase activity (false discovery rate was 0.04021511), ligase activity, and forming aminoacyl-tRNA and related compounds (false discovery rate was 0.04668625). These five processes were directly or indirectly related to mRNA transcription and translation. With regard to the processes of DNA metabolic process (false discovery rate was 0.001424074), meiosis (false discovery rate was 0.004201701), G2/M transition of mitotic cell cycle (false discovery rate was 0.0119566168), and positive regulation of sister chromatid cohesion (false discovery rate was 0.0119566168), these were directly related to soybean metabolism, 1-phosphatidylinositol 4-kinase activity (false discovery rate was 0.000563865), and phosphatidylinositol-mediated signaling (false discovery rate was 0.040720139), These in turn were directly related to membrane permeability. The above results indicate that these key sRNAs can directly participate in and regulate the metabolic level of soybeans to resist the pathogenicity of *P. sojae* and can regulate the pathway of *Phytophthora* infection by changing the membrane permeability.

#### Functional Enrichment Analysis of Targeted *Phytophthora sojae*

To further explore the regulatory effects of differentially expressed soybean sRNA on *P. sojae*, the 1,683 *P. sojae* mRNA obtained in this paper was first converted into a format recognized by the String database according to the String annotation conversion file (67,593.protein.aliases.v11.0). Subsequently, the converted sequence file was imported into the String database, and the overall network PPI enrichment *p*-value obtained was 1.0e−16. Among them, the *p*-value of 11 KEGG_Pathway was less than the credible threshold value of 0.05, as shown in Table 7.

**Table 7:** Soybean resistance to *Phytophthora* infestation sRNA targeting *Phytophthora sojae* KEGG_Pathway.

Pathway	Description	False discovery rate	Number of genes
psoj03013	RNA transport	0.00033	8
psoj03008	Ribosome biogenesis in eukaryotes	0.0012	6
psoj03420	Nucleotide excision repair	0.0067	4
psoj00562	Inositol phosphate metabolism	0.0236	4
psoj03018	RNA degradation	0.0236	4
psoj04070	Phosphatidylinositol signaling system	0.0236	4
psoj01100	Metabolic pathways	0.0333	17
psoj03030	DNA replication	0.0333	3
psoj04145	Phagosome	0.0358	3
psoj00561	Glycerolipid metabolism	0.0489	3
psoj04144	Endocytosis	0.0489	4

As can be observed from Table 7, the key soybean disease-resistant sRNA can participate in and regulate various regulatory pathways of *P. sojae*, principally including *P. sojae* transmembrane transport, nucleotide excision, repair, and transportation processes. A finding was that differentially expressed sRNA can possess a regulatory function in transmembrane transport and the exercise of normal functions of *P. sojae*. In particular, the pathways directly linked to the entry of soybean sRNA into *P. sojae* cells through transmembrane transport were inositol phosphate metabolism (false discovery rate was 0.0236), phosphatidylinositol signaling system (false discovery rate was 0.0236), glycerolipid metabolism (false discovery rate was 0.0489), and endocytosis (false discovery rate was 0.0489). The pathways that inhibited the function of mRNA through shearing and degradation with soybean sRNA include RNA transport (false discovery rate was 0.00033), nucleotide excision repair (false discovery rate was 0.0067), RNA degradation (false discovery rate was 0.0236), and phagosome (false discovery rate was 0.0358). In accordance with the above analysis, an observation can be made from the data that the key soybean disease-resistant sRNA can completely regulate the permeability of the *P. sojae* membrane and provide an environment therefore to enter the *P. sojae* of active reverse infection. A further observation can be made that the key soybean disease-resistant sRNA can completely regulate the permeability of the *P. sojae* membrane and provide an environment to enter the active reverse infection of *P. sojae*. On the other hand, the key sRNA of soybean disease resistance can completely affect the pathogenicity of *P. sojae* by removing and degrading the mRNA of *P. sojae*.

#### Summaries of Functional Enrichment Analysis

The results indicate that the differentially expressed soybean sRNA can regulate soybean metabolism, ligase, translation factor activity, transmembrane transport, and other biological processes related to disease resistance. Further, said sRNA can regulate the processes of *P. sojae* endocytosis, mRNA shearing and translation, and transmembrane transport. These processes are the central processes of soybean sRNA transboundary regulation of *P. sojae*. This chapter verifies that the selected 768 differentially expressed sRNAs are the key sRNAs for soybean disease resistance.

### Comparison and Analysis of Models Based on Optimized Parameters

**Figure 11:**
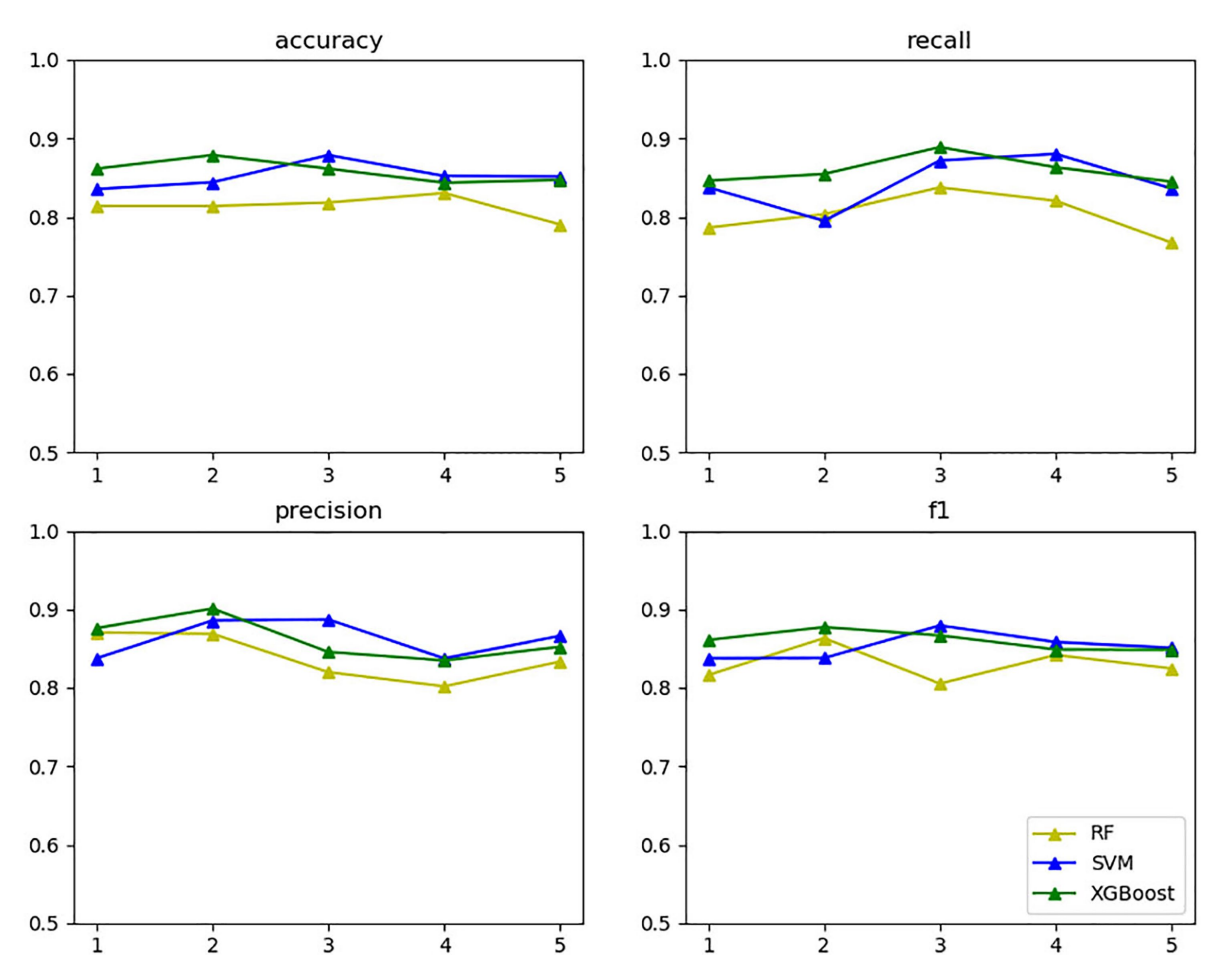
Three model evaluations.

In the vein of comparing the above three models’ classification effects, 5-fold cross-validation was performed on the training set for the models with the selected optimal parameters, and statistics were calculated on the accuracy, recall, precision, and F1 value thereof, as exhibited in Figure 11.

The yellow, blue, and green lines in the above figure’s upper right label correspond to random forest, SVM, and XGBoost, respectively. The four subgraphs respectively represent the accuracy, recall, precision, and F1 value of the above algorithm for five cross-validations on the training set. As the above figure demonstrates, XGBoost and SVM performed well in all aspects.

The reason is that based on support vectors, SVM can avoid the complexity of high-dimensional space and can effectively classify small data samples. Both the number of samples and the number of sample features are suitable for this data set. The disadvantage is that it is not effective for multi-classification problems and large sample data, but the data in this article are a small sample data and a two-classification problem. Random forest builds multiple classifier decision trees by randomly extracting samples and features. Only when more than half of the base classifiers have errors will they make incorrect predictions and handle high-dimensional data well. The disadvantage is that it will overfit in some noisy classification or regression problems, but the data in this article do not match it. It may not produce a good classification for small data or low-dimensional data (data with fewer features). Since this article is a small data sample, the effect will be relatively poor. XGBoost uses parallel optimization on feature granularity, adds a regularization term to the objective function to prevent overfitting, and draws on the advantages of random forest support column sampling, which not only reduces overfitting but also reduces the complexity of calculation. At the same time, XGBoost can perform the next iteration process after completing one iteration; that is, the subsequent iteration process contains the predicted value of the previous iteration process, so it has a better classification effect for the data set of this article. The disadvantage is that the space complexity of the presorting process is too high and the execution speed is slower than random forest (bagging), but this article does not have too many requirements for these.

Receiver operating characteristic-area under the curve (ROC-AUC) was adopted to evaluate the model further. The ROC curve used the true-positive rate (TPR) as the ordinate and the false-positive rate (FPR) as the abscissa. The TPR is the recall rate mentioned above. The calculation method of the FPR is shown in Eq. 8:


(8) $$\mathrm{FPR}=\frac{\mathrm{FP}}{\mathrm{TN}+\mathrm{FP}}$$

**Figure 12:**
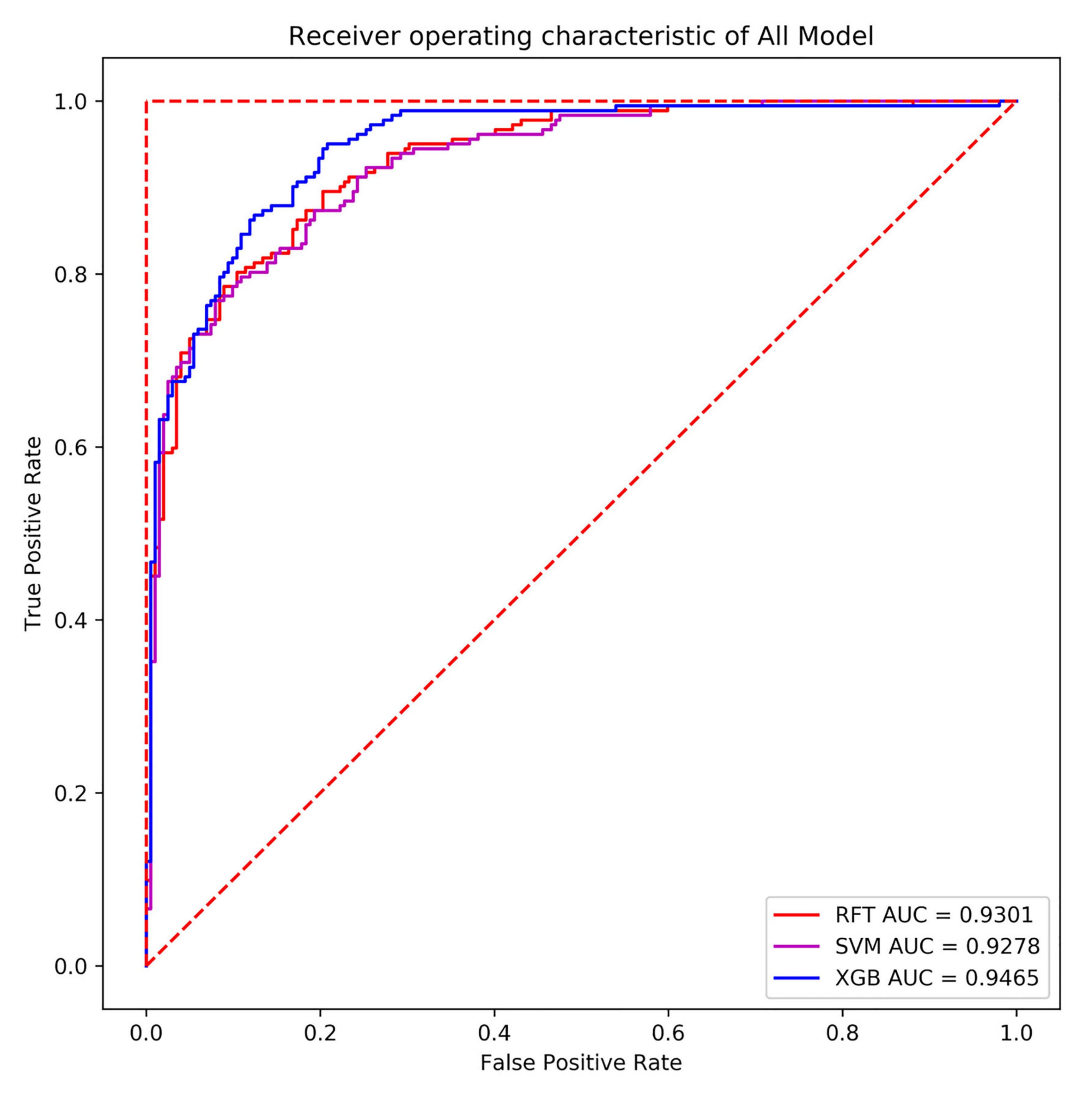
Receiver operating characteristic (ROC) curves of the three models.

In terms of the ROC curve, the (0, 1) point in the upper left corner, that is, the point where the FPR was 0 and the TPR was 1, indicates that FN was 0 and FP was 1, implying that all samples were correctly classified. The (0, 0) point in the lower left corner, specifically, the point where the FPR was 0 and the TPR was 0 implies that FP was 0 and TP was 0, indicating that the classifier predicted all samples as negative samples. In parallel, the (1, 1) point in the upper right corner reveals that the classifier predicted all samples as positive samples. The (1, 0) point in the lower right corner, that is, the point where the FPR was 1 and the TPR was 0, means that the classifier misclassified all samples, that is, predicted all positive samples as negative samples and predicted all negative samples as positive samples. On the diagonal, FP = TN and TP = FN, namely, random classification. That is, the closer the overall trend was to the (0, 1) point, the more superior the model effect. In many cases, the ROC curve does not clearly indicate which classifier performs better. For this reason, the area under the ROC curve and the coordinate axis were used, in other words, AUC as the evaluation criterion, to compare the models more accurately. Figure 12 exhibits the ROC curves of the random forest, SVM, and XGBoost model verification and evaluation using 384 verification set data.

From the above figure, a finding can be made that the areas under the three machine learning models’ curves were relatively large, the curves were relatively smooth overall, and no overfitting phenomenon occurred. In the present paper, XGBoost with the highest AUC value was selected as the final classifier. Meanwhile, the classifier also performed well in terms of accuracy and recall. The accuracy thereof on the verification set was 86.98%, and the AUC value was 0.9465. In the training set, the AUC value was 0.9805, implying that the overall effect of the model was better. Hence, the model can also predict the differential expression of *P. sojae* sRNA mapped to the *P. sojae* genome after infection and has reference significance for the selection of differentially expressed sRNAs in plants infected by other types of fungi.

## Discussion

### Limitations of Target Gene Prediction

In terms of differentially expressed sRNAs, because of the long running time, only TAPIR was utilized as a tool to predict the target genes thereof, upon which functional enrichment analysis was directly performed on these target genes. For plant-related target gene tools, psRNATarget and TAPIR are more commonly used. Concerning other target gene prediction software, the algorithms therein are different. Only TAPIR was adopted in the present article, meaning that the results obtained may not be complete. A better prediction effect may be produced if a variety of related software is employed.

### Discover the Significance of Biological Processes and Regulatory Pathways

The present study found that the differentially expressed sRNAs of soybean can regulate multiple disease-resistant and growth-related biological processes of soybean itself and the core processes of multiple *Phytophthora sojae*. The present research has laid the foundation for the potential of other plants to resist fungal infection. Further, when various plants resist fungal infection, there are certain similarities between the biological processes and regulatory pathways thereof that are worthy of discussion and research. If the connection between these processes can be discovered, this will provide crucial theoretical basis and new control ideas for the control of plant diseases.

### Model Universality

XGBoost was selected as the final classifier in the present paper, which has a good predictive effect for predicting the correlation between unknown soybean sRNA sequence and resistance to *P. sojae* infection. This provides theoretical guidance for the conduct of biological experiments and reduces experimental costs. Be that as it may, whether this model is applicable to other plants and fungi-based resistance analysis based on sRNA remains undetermined and requires further study. With sufficient data, the key sRNAs for plant antifungal disease can be predicted premised on the data analysis and model construction methods proposed in the present article to test the model’s universality.

### Application of Machine Learning Models

The present article utilized only random forest, SVM, and XGBoost, which are the three methods to construct a sRNA prediction model for soybean resistance to *Phytophthora* pathogenicity. There are numerous models in machine learning that can also classify samples, using more models potentially finding better prediction results.

## Data Availability Statement

The original contributions presented in the study are included in the article/supplementary material, further inquiries can be directed to the corresponding author.

## Author Contributions

LD and YL conceived and directed the project. JC and SS obtained the raw data and interpreted the data. LD, JC, SS, HaZ, and HeZ conducted the data analysis and interpreted the results. JC and SS helped to design the study and reviewed the data. LD, JC, and HaZ wrote and/or edited the manuscript. All the authors have drafted and reviewed the manuscript and approved it for publication.

### Conflict of Interest

The authors declare that the research was conducted in the absence of any commercial or financial relationships that could be construed as a potential conflict of interest.

## Footnotes

^1^ https://hpc.nih.gov/apps/sratoolkit.html

^2^ https://cutadapt.readthedocs.io/en/stable/

^3^ http://www.bioinformatics.babraham.ac.uk/projects/fastqc/

^4^ https://sourceforge.net/projects/bowtie-bio/files/bowtie2/2.3.4.1/

^5^ http://www.htslib.org/download/

^6^ http://bioinformatics.psb.ugent.be/webtools/tapir/

^7^ https://string-db.org/

^8^ https://www.soybase.org/

^9^ http://rna.tbi.univie.ac.at/cgi-bin/RNAWebSuite/RNAfold.cgi
