# Corrigendum: Research on the mechanism of soybean resistance to *Phytophthora* infection using machine learning Methods

**DOI:** 10.3389/fgene.2022.1062928

**Published:** 2022-10-24

**Authors:** Junxia Chi, Shizeng Song, Hao Zhang, Yuanning Liu, Hengyi Zhao, Liyan Dong

**Affiliations:** ^1^ College of Software, Jilin University, Changchun, China; ^2^ Key Laboratory of Symbolic Computation and Knowledge Engineering of Ministry of Education, Jilin University, Changchun, China; ^3^ College of Computer Science and Technology, Jilin University, Changchun, China

**Keywords:** sRNA data analysis, differential expression, machine learning, resistance mechanism, *Phytophthora sojae*

In the published article, the **affiliation** of “College of Software, Jilin University, Changchun, China” was erroneously omitted and the authors’ affiliations had incorrect attributions as a result.

The authors apologize for this error and state that this does not change the scientific conclusions of the article in any way. The original article has been updated.

